# Strong interlayer interactions in bilayer and trilayer moiré superlattices

**DOI:** 10.1126/sciadv.abk1911

**Published:** 2022-03-25

**Authors:** Saien Xie, Brendan D. Faeth, Yanhao Tang, Lizhong Li, Eli Gerber, Christopher T. Parzyck, Debanjan Chowdhury, Ya-Hui Zhang, Christopher Jozwiak, Aaron Bostwick, Eli Rotenberg, Eun-Ah Kim, Jie Shan, Kin Fai Mak, Kyle M. Shen

**Affiliations:** 1 Department of Physics, Laboratory of Atomic and Solid State Physics, Cornell University, Ithaca, NY, USA.; 2 Department of Materials Science and Engineering, Cornell University, Ithaca, NY, USA.; 3 Kavli Institute at Cornell for Nanoscale Science, Ithaca, NY, USA.; 4 School of Applied and Engineering Physics, Cornell University, Ithaca, NY, USA.; 5 Department of Physics, Harvard University, Cambridge, MA, USA.; 6 Advanced Light Source, E. O. Lawrence Berkeley National Laboratory, Berkeley, CA, USA.

## Abstract

Moiré superlattices constructed from transition metal dichalcogenides have demonstrated a series of emergent phenomena, including moiré excitons, flat bands, and correlated insulating states. All of these phenomena depend crucially on the presence of strong moiré potentials, yet the properties of these moiré potentials, and the mechanisms by which they can be generated, remain largely open questions. Here, we use angle-resolved photoemission spectroscopy with submicron spatial resolution to investigate an aligned WS_2_/WSe_2_ moiré superlattice and graphene/WS_2_/WSe_2_ trilayer heterostructure. Our experiments reveal that the hybridization between moiré bands in WS_2_/WSe_2_ exhibits an unusually large momentum dependence, with the splitting between moiré bands at the Γ point more than an order of magnitude larger than that at *K* point. In addition, we discover that the same WS_2_/WSe_2_ superlattice can imprint an unexpectedly large moiré potential on a third, separate layer of graphene (g/WS_2_/WSe_2_), suggesting new avenues for engineering two-dimensional moiré superlattices.

## Teaser

Transition metal dichalcogenide heterostructures show strong interactions and can imprint a moiré potential to a separate layer.

## INTRODUCTION

Moiré superlattices composed of stacked two-dimensional materials present a versatile platform for engineering and investigating new quantum states of matter (*1*–*17*). Moiré superlattices are formed when two monolayers are stacked together with a twist angle and/or a lattice mismatch. As a result of these superlattices, new and distinct “minibands” associated with the smaller moiré Brillouin zone are formed (*18*). Because the bandwidth of these new moiré minibands can be narrow, new emergent properties arising from strong Coulomb interactions can be realized and controlled in these systems (*1*, *6*, *7*). In particular, transition metal dichalcogenide (TMD)–based moiré superlattices have demonstrated a series of emergent phenomena, including moiré excitons (*3*, *4*, *16*), flat bands (*17*), and correlated insulating states (*6*–*8*). Because of the quasiperiodic nature of moiré superlattices, their precise electronic structure over a wide range of energy and momentum space is still not well understood and can only be treated by various approximate methods (*18*–*21*). Experimental probes such as transport and optical spectroscopy are generally only sensitive to the moiré electronic structure in a very narrow range of energy and momentum space around the lowest-lying states, typically near the *K* point for graphene and monolayer TMDs. To achieve a deeper fundamental understanding of moiré superlattices, it is therefore critical to reveal their electronic structure across a broad range of energies and momentum space and to understand the mechanisms by which moiré superpotentials can be generated and imposed.

**Fig. 1.**
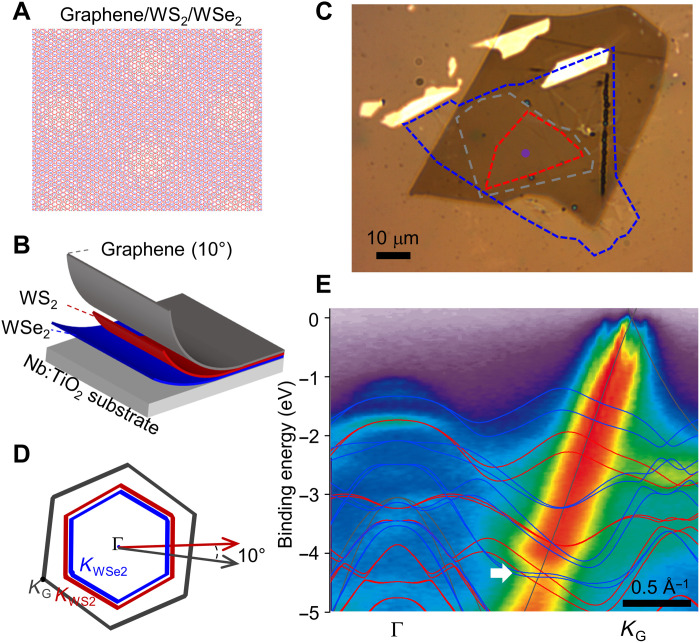
Moiré superlattices of graphene/WS_2_/WSe_2_. (**A**) Schematic atomic arrangement of an aligned WS_2_/WSe_2_ moiré superlattice with a graphene layer, where WS_2_, WSe_2_, and graphene layers are in red, blue, and gray, respectively. (**B** and **C**) Schematics (B) and optical micrograph (C) of a moiré superlattice sample on a Nb:TiO_2_ substrate, where WS_2_, WSe_2_, and graphene are represented by red, blue, and gray colors, respectively. (**D**) Schematic of Brillouin zones of WS_2_ (red), WSe_2_ (blue), and graphene (gray). (**E**) ARPES spectra taken along Γ-*K*_G_, with overlaid density functional theory (DFT) calculations of the band structures for individual monolayer graphene (gray), WS_2_ (red), and WSe_2_ (blue). The white arrow indicate hybridization between the graphene and TMD bands.

Here, we use angle-resolved photoemission spectroscopy (ARPES) with submicron spatial resolution to address these questions by investigating two types of moiré superlattices. In an aligned WS_2_/WSe_2_ heterostructure, where a moiré potential with a wavelength of 8 nm emerges (Fig. 1A), we observe distinct minibands near Γ, with an unexpectedly large energy splitting of 220 meV between the first and second bands, much larger than the energy splitting of ~15 meV at the valence band maximum (*K* point) (*22*). We also reveal that, in a trilayer heterostructure of graphene/WS_2_/WSe_2_ (g/WS_2_/WSe_2_), the aligned WS_2_/WSe_2_ heterostructure can imprint an unexpectedly large moiré potential on the graphene layer (Fig. 1B), presenting a new avenue for engineering minibands in quasiperiodic structures.

## RESULTS

In Fig. 1 (B and C), we show the schematic and an optical micrograph of a g/WS_2_/WSe_2_ moiré superlattice sample investigated in this study. In this heterostructure, the WS_2_ and WSe_2_ are aligned and are both 10°-twisted with respect to the graphene layer (Brillouin zone schematically shown in Fig. 1D). Because of the different sizes of the constituent flakes, regions of with different kinds of heterostructures exist, as shown by the outlines. Because of the small size of the beam spot (<1 μm^2^), the center region of g/WS_2_/WSe_2_ moiré superlattice can be identified and investigated. Figure 1E shows an ARPES cut along Γ-*K*_G_ (graphene *K* point), together with overlaid density functional theory (DFT) calculations of the band structures for individual monolayer graphene, WS_2_, and WSe_2_. The hybridization between the graphene and TMD layers can be observed by the formation of distinct gaps where their bands intersect, as shown by the white arrow in Fig. 1E.

**Fig. 2.**
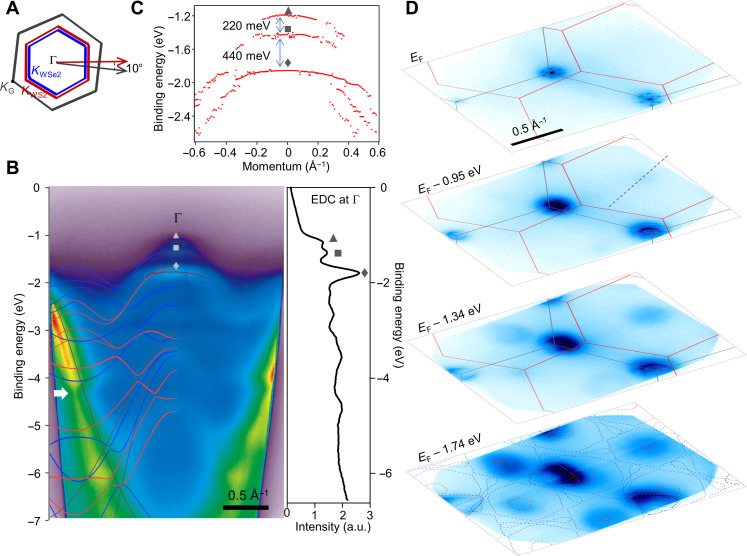
Direct observation of minibands in a graphene/WS_2_/WSe_2_ moiré superlattice. (**A**) Schematic of Brillouin zones of graphene (gray), WS_2_ (red), and WSe_2_ (blue). (**B**) An ARPES cut across Γ point along the purple dashed line shown in (D), where the left half is overlaid with the bands calculated by DFT (gray, graphene; red, WS_2_; blue, WSe_2_). Right: Energy distribution curve (EDC) taken at Γ, and the moiré bands at different increasing binding energies are labeled with a triangle, a square, and a diamond, respectively. The fitted peak positions are further indicated with red dots in the ARPES cut. a.u., arbitrary units. (**C**) Extracted EDC peak positions showing the large energy splittings between the three bands observed at Γ. (**D**) Constant energy contours at various binding energies. The gray and red lines in the top three panels indicate the Brillouin zones of graphene and WSe_2_, respectively. Bottom: Dashed lines show the bands produced by DFT calculations: gray for graphene, red for WS_2_, and blue for WSe_2_.

The band structures of WS_2_ and WSe_2_ are qualitatively similar, with their valence band maxima both located at their respective *K* points (*K*_TMD_), which are hundreds of millielectron volts above the valence band at Γ [e.g., 500 meV for WSe_2_ (*23*)]. In the aligned WS_2_/WSe_2_ superlattice, the existence of moiré minibands and excitons has been the subject of extensive investigation by optical and transport measurements (*3*–*7*, *16*), which probes the states solely around the valence band maximum at *K*_TMD_, and hence, their behavior throughout momentum space remains an open question. In Fig. 2B, we show ARPES spectra from g/WS_2_/WSe_2_ (Brillouin zone schematically shown in Fig. 2A); at the Γ point, the lowest-lying graphene bands are deep in energy (~3 eV), so the relevant near-*E*_F_ (*E*_F_ denotes the Fermi level) electronic states belong only to WS_2_/WSe_2_. In a purely nonhybridized scenario, WSe_2_ and WS_2_ would individually contribute only a single band at Γ, whereas in Fig. 2 (B and C), three bands can be clearly observed (with minimum binding energies of 1.16, 1.38, and 1.80 eV, respectively), signifying hybridization between the WS_2_ and WSe_2_ layers. By comparing the binding energies of these three bands with those of single-layer WSe_2_ (*23*) and WS_2_ (*24*), we identify the deepest of the three bands (at 1.80 eV) as primarily of WS_2_ character and the middle and lowest-lying moiré bands to be of primarily WSe_2_ character. The energy splitting at Γ between the lowest-lying miniband and the middle band, Δ*E* = 220 ± 5 meV, is notably large, more than an order of magnitude larger than the energy splitting between the lowest-lying moiré minibands at *K*_TMD_ [15 meV; (*22*)].

**Fig. 3.**
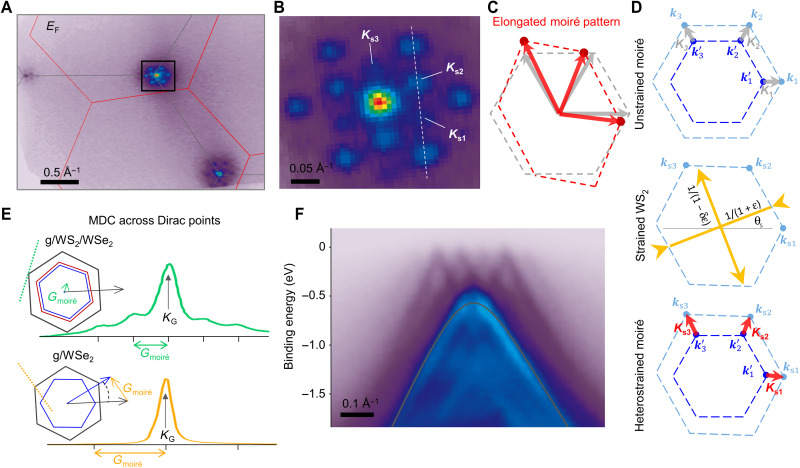
Imprinting effect of moiré superlattice in a graphene/WS_2_/WSe_2_ heterostructure. (**A** and **B**) Constant energy contours (A) and an enlarged area (B) near *K*_G_ at *E*_F_, showing multiple replica Dirac points separated by the WS_2_/WSe_2_ moiré wavevectors. (**C**) Undistorted (gray) and elongated (red) hexagon pattern of the replica Dirac points. (**D**) Top: Construction of undistorted moiré wavevector (*K*_1_, *K*_2_, and *K*_3_, represented by gray arrows) from reciprocal wavevectors of WS_2_ (*k*_1_, *k*_2_, and *k*_3_, represented by light blue spots) and WSe_2_ (*k*′_1_, *k*′_2_, and k′_3_, represented by blue spots). Middle: Reciprocal wavevectors of WS_2_ under a uniaxial strain of ε, along the direction θ_s_ with respect to the *x* axis. Bottom: Moiré wavevectors in heterostrained WS_2_/WSe_2_ moiré superlattice (*K*_s1_, *K*_s2_, and *K*_s3_, represented by red arrows). (**E**) Momentum distribution curves (MDCs) taken across the Dirac point along the respective *G*_moiré_ direction in g/WS_2_/WSe_2_ and g/WSe_2_, as schematically shown by the dashed lines in the insets, and replica is only observed in the former heterostructure. (**F**) An ARPES cut taken across multiple replica Dirac cones, along the white dashed line indicated in (B), where the dark gray line represents original monolayer graphene bands calculated by DFT.

We believe that the unusually large momentum dependence of the splitting of the moiré minibands arises from the momentum dependence of the orbital character of the transition metal *d* states. The TMD *d* orbitals have a primarily in-plane character at *K*_TMD_ but have substantially more out-of-plane character at Γ, which facilitate stronger interlayer hybridization and stronger moiré interactions at Γ. We note that, in a previous report of MoSe_2_/WSe_2_, a large splitting of 200 meV was observed at Γ between the WSe_2_ valence band and the hybridized heterobilayer band. This large splitting was hypothesized to arise from commensuration between the MoSe_2_ and WSe_2_ layers, which are very closely matched (Δ*a*/*a* ~ 0.3%) (*23*). On the other hand, in the case of WS_2_ and WSe_2_, the lattice mismatch is more than one order of magnitude larger (*Δa/a* ~ 4%). Such a large lattice mismatch leads to elastic energy of more than two orders of magnitude higher to form commensurate domains compared to that in MoSe_2_/WSe_2_ superlattices, making it unlikely that the WS_2_ and WSe_2_ could be commensurate. In addition, data from Fig. 3 definitively rule out the possibility of commensuration, as discussed later. Another signature of the moiré superlattice is that the bands around Γ are significantly flatter than expected from individual WSe_2_ or WS_2_ monolayers, potentially due to zone folding from the small moiré Brillouin zone. For instance, the effective mass for the middle band at Γ in g/WS_2_/WSe_2_ is approximately m* = 4.1 *m*_0_, as opposed to only m* = 1.4 *m*_0_ in the absence of TMD moiré superlattice (i.e., g/WSe_2_). Given the large energy splittings observed here (i.e., hundreds of millielectron volts), it may be possible to construct moiré superlattices with emergent electronic states surviving to room temperature, for instance, in indirect-gap heterostructures such as moiré superlattices between two individual TMD bilayers, where the valence band maximum is at Γ instead of *K*.

Up to this point, we have focused on the moiré minibands at Γ where the low-energy orbitals arise purely from the WS_2_ and WSe_2_ layers, making any hybridization effects with the graphene layer largely irrelevant. However, when the graphene bands approach *E*_F_, the effects of any interactions between the graphene bands and the underlying moiré superpotential should become pronounced. As shown in Figs. 2D and 3 (A and B), interactions between the moiré superlattice potential and the Dirac electrons in the graphene layer at *K*_G_ give rise to multiple replica Dirac points, which are separated by the WS_2_/WSe_2_ moiré wavevector (*G*_moiré_ = 0.07 Å^−1^), where the intensity of the first-and second-order replicas is >30% of the original band and even third-order replicas are evident. An idealized triangular moiré superlattice should result in a perfectly hexagonal pattern of replica Dirac points, but instead, we observe a sizeable distortion of this hexagonal pattern (an elongation along one axis by 21 ± 2%) in the data of Fig. 3 (A and B). This elongation is more than an order of magnitude larger than our experimental momentum uncertainty of Δ*k* = 1.4%, determined by comparing the separation and angles between different graphene Dirac points (1.678 and 1.703 Å^−1^, respectively) to its known lattice constant (1.703 Å^−1^). This large distortion (schematically shown in Fig. 3C) can be generated by a very modest uniaxial strain (~0.7%) in one of the layers relative to the other, as illustrated in Fig. 3D (also see the Supplementary Materials). Unstrained WS_2_ and WSe_2_ lattices lead to three moiré wavevectors with the same length, rotated by 60° with respect to each other, and a small uniaxial strain in one layer will lead to a greatly amplified distortion of those moiré wavevectors, as shown schematically in Fig. 3 (C and D, bottom). While distorted moiré superlattices have also recently been reported by atomically resolved scanning tunneling microscopy experiments (*25*), our ARPES measurements provide the first imaging of this effect using a momentum-resolved spectroscopic probe over micron length scales. Furthermore, the observation of a finite moiré wave vector conclusively rules out commensuration between WS_2_/WSe_2_ as the origin for the large splitting of the moiré minibands at Γ observed in Fig. 2 (B and C).

While it is possible that these replicas could also potentially arise from final state photoelectron diffraction effects (*26*–*30*), we believe that the replicas observed here more likely arise from intrinsic interactions between the graphene layer and the moiré superlattice. First, we do not observe replica Dirac cones in the g/WSe_2_ heterostructure (Fig. 3E, data of g/WSe_2_ taken at a different region on the same sample), as would also be expected if final state effects played a critical role. Second, the presence of energy gaps between the graphene and WSe_2_ and WS_2_ bands (noted by the white arrow in Fig. 2B) indicates strong intrinsic interactions between the different layers. Although we cannot directly observe clear energy gaps between the different replica Dirac cones themselves (Fig. 3F), we suspect that this is most likely due to the combination of the large thermal broadening (*T* = 300 K) and experimental energy resolution (Δ*E* = 125 meV), which would likely obscure any small gaps caused by the moiré superpotential.

These Dirac moiré replicas suggest a new pathway toward imprinting moiré superlattice potentials in layered heterostructures. In conventional bilayer moiré superlattices, the size and orientation of the moiré wavevector is entirely determined by the twist angle and lattice mismatch between the two layers. On the other hand, for moiré superlattices that are imprinted onto a third layer, the moiré wavevector becomes an adjustable parameter determined solely by the composition of the moiré bilayer, is independent of the third layer, and thus should allow for a broader range of possible moiré superlattices and designer electronic materials that can be realized, such as long-wavelength superlattices in materials that do not have a close lattice match. Moiré superlattices consisting of more than two layers have demonstrated its tunability such as superconductivity in twisted trilayer graphene systems (*31*, *32*). In conventional moiré superlattices, the spatial variations in hybridization are known to be caused by changes in the local stacking between atoms (*33*). On the other hand, the mechanism by which a moiré superlattice is imprinted onto a third material remains an open question and is unlikely to be due to spatial variations in local stacking, because in “imprinted” structures, different sites separated by a moiré wavelength may have very different local atomic structures and stacking. This suggests that other effects, such as spatially modulated charge transfer between WS_2_/WSe_2_ and graphene and/or topography, may be more relevant for determining the strength of the superlattice potential in imprinted moiré superlattices.

## DISCUSSION

By investigating moiré superlattices over a wider range of energies and momenta than possible by optical or transport probes, our experiments have revealed microscopic structures of moiré superlattices and that moiré superlattice effects are richer than previously known, motivating a new understanding of the mechanism by which these moiré potentials are formed. In addition, our work reveals a novel approach to construct new kinds of superlattices by imprinting strong superpotentials with arbitrary and controllable wavelengths from an existing moiré superlattice onto a third, separate layer.

## MATERIALS AND METHODS

### Sample fabrication

Atomically thin flakes were exfoliated from bulk crystals onto silicon substrates with 285-nm oxides. Monolayer WSe_2_ and WS_2_ were identified by their optical contrast. The crystal orientations of WSe_2_ and WS_2_ and the twist angle of 60° between WS_2_ and WSe_2_ (fig. S2) were determined by angle-resolved optical second-harmonic generation (SHG). A pulsed laser with a duration of 100 fs at 800 nm was used as the excitation source, and the cross-polarized SHG signal was collected at 400 nm. A sixfold symmetry of the SHG signal was resolved by rotating the excitation polarization with respect to the sample orientation. The heterostructure was assembled using a layer-by-layer dry transfer technique described in (*34*). The thin flakes were picked up, one by one, with a stamp consisting of a polycarbonate thin film attached to a polydimethylsiloxane. Monolayer graphene and few layer hBN were used as the top and bottom encapsulating layers, respectively. The zigzag direction of the WSe_2_ and WS_2_ monolayers was aligned by SHG. The completed heterostructure was released onto the conducting, 0.5 weight % Nb-doped rutile TiO_2_ (100) substrate by heating the stamp to 180°C. The heterostructure was then rinsed in chloroform and isopropyl alcohol to remove the melted polycarbonate film and cleaned using an atomic force microscope tip in the contact mode.

### ARPES measurements

After assembly and cleaning, the samples were sealed in a vacuum chamber inside an inert gas glovebox and then pumped to 10^−5^ torr before transportation and loading into the Microscopic and Electronic Structure Observatory ultra-high vacuum facility at beamline 7.0.2 of the Advanced Light Source in Berkeley. The samples were annealed at 300°C for 10 hours before ARPES measurements. ARPES data were collected using a hemispherical Scienta R4000 electron analyzer with the energy and momentum resolutions set to 125 meV and 0.015 Å^−1^, respectively. The photon energy was set to 70 eV with the incident beam focused using an x-ray capillary to a nominal spot size of ~1 μm. Samples were maintained at room temperature throughout measurements.

### Density functional theory

DFT calculations of freestanding graphene, WSe_2_, and WS_2_ were performed using the Perdew-Burke-Ernzerhof generalized gradient functional (*35*), as implemented in the Quantum ESPRESSO suite (*36*, *37*). Full and scalar relativistic projector augmented wave (*38*) pseudopotentials from the PSlibrary (*39*) were used for WSe_2_/WS_2_ and graphene, respectively. For WSe_2_ and WS_2_, an energy cutoff of 120 rydberg (Ry) (500 Ry) was used for the wave function (charge density), and a Γ-centered 16 × 16 × 3 Monkhorst-Pack (MP) mesh (*40*) was used for *k*-space discretization. For graphene, an energy cutoff of 150 Ry (800 Ry) was used for the wave function (charge density) along with a Γ-centered 21 × 21 × 5 MP mesh. The calculated graphene Dirac cones are renormalized to match the experimentally observed Fermi velocity of *v*_F_ = 1.13 × 10^6^ m s^−1^. The DFT calculations for WSe_2_/WS_2_ (graphene) were then downfolded onto a manifold of 22 (*18*) maximally localized Wannier functions (*41*, *42*) using the Wannier90 package (*43*) for interpolation onto the same *k*-point mesh as the ARPES data and for construction of the constant energy contours.
