# Supplementary material for: Strong interlayer interactions in bilayer and trilayer moiré superlattices
Source: Sci Adv. 2022 Mar 25;8(12):eabk1911. doi: 10.1126/sciadv.abk1911 (PMC8956267; doi:10.1126/sciadv.abk1911)
Supplement: Supplementary file 1 — Supplementary Text Figs. S1 and S2 [file sciadv.abk1911_sm.pdf]

Science Advances
AAAS

Supplementary Materials for

Strong interlayer interactions in bilayer and trilayer moiré superlattices

Saien Xie*, Brendan D. Faeth, Yanhao Tang, Lizhong Li, Eli Gerber, Christopher T. Parzyck, Debanjan Chowdhury, Ya-Hui Zhang, Christopher Jozwiak, Aaron Bostwick, Eli Rotenberg, Eun-Ah Kim, Jie Shan, Kin Fai Mak, Kyle M. Shen*

*Corresponding author. Email: sx68@cornell.edu (S.X.); kmshen@cornell.edu (K.M.S.)

Published 25 March 2022, *Sci. Adv.* **8**, eabk1911 (2022)
DOI: 10.1126/sciadv.abk1911

This PDF file includes:

Supplementary Text
Figs. S1 and S2

Supplementary Text

Moiré superlattice under uniaxial heterostrain

The layered materials in moiré superlattice samples are often under strain, which can be introduced during the sample preparation process. Such strain affects the reciprocal wavevector of the corresponding material, and therefore the moiré wavevectors. In the case where both layers are strained equally, the change in moiré wavevector is minimal (given the magnitude of strain is typically less than 1%). In contrast, when the two layers are strained differently (i.e., heterostrain), the moiré wavevector can have significant changes. In the following discussion, we focus on heterostrained WS_2/WSe_2 superlattices, where WS_2 is under a uniaxial strain while WSe_2 remains unstrained.

We assign $\boldsymbol{k_1}$, $\boldsymbol{k_2}$, and $\boldsymbol{k_3}$ to be the reciprocal wavevectors of WS_2, and $\boldsymbol{k'_1}$, $\boldsymbol{k'_2}$, and $\boldsymbol{k'_3}$ to be the reciprocal wavevectors of WSe_2.

$$\boldsymbol{k_1} = \begin{pmatrix} k_{\mathrm{WS2}} \\ 0 \end{pmatrix}, \boldsymbol{k_2} = \begin{pmatrix} k_{\mathrm{WS2}} \times cos(60) \\ k_{\mathrm{WS2}} \times sin(60) \end{pmatrix}, \boldsymbol{k_3} = \begin{pmatrix} k_{\mathrm{WS2}} \times cos(120) \\ k_{\mathrm{WS2}} \times sin(120) \end{pmatrix} \ and\ k_{\mathrm{WS2}} = \frac{4\pi}{\sqrt{3} * a_{WS2}}$$

$$\boldsymbol{k'_1} = \begin{pmatrix} k_{\mathrm{WSe2}} \\ 0 \end{pmatrix}, \boldsymbol{k'_2} = \begin{pmatrix} k_{\mathrm{WSe2}} \times \cos(60) \\ k_{\mathrm{WSe2}} \times \sin(60) \end{pmatrix}, \boldsymbol{k'_3} = \begin{pmatrix} k_{\mathrm{WSe2}} \times \cos(120) \\ k_{\mathrm{WSe2}} \times \sin(120) \end{pmatrix} \ and\ k_{\mathrm{WSe2}} = \frac{4\pi}{\sqrt{3} * a_{WSe2}}$$

The moiré wavevectors (shown in Fig. S1A) are then given by

$$\boldsymbol{K_1} = \boldsymbol{k_1} - \boldsymbol{k'_1}, \boldsymbol{K_2} = \boldsymbol{k_2} - \boldsymbol{k'_2}, \boldsymbol{K_3} = \boldsymbol{k_3} - \boldsymbol{k'_3}$$

A uniaxial strain with the magnitude of ε, along the direction of θ_s respective to the x axis, which can be described by the following matrix:

$$\boldsymbol{S}(\varepsilon,\theta_s) = \begin{pmatrix} \cos(\theta_s) & \sin(\theta_s) \\ -\sin(\theta_s) & \cos(\theta_s) \end{pmatrix} \begin{pmatrix} \frac{1}{1+\varepsilon} & 0 \\ 0 & \frac{1}{1-\delta\varepsilon} \end{pmatrix} \begin{pmatrix} \cos(\theta_s) & -\sin(\theta_s) \\ \sin(\theta_s) & \cos(\theta_s) \end{pmatrix}$$

Where δ is the Poisson ratio of the material being strained ($\delta = 0.22$ for WS_2).

The reciprocal wavevectors of strained WS_2 (Fig. S1B) become

$$\boldsymbol{k_{s1}} = \boldsymbol{S}(\varepsilon,\theta_s)\,\boldsymbol{k_1}, \boldsymbol{k_{s2}} = \boldsymbol{S}(\varepsilon,\theta_s)\,\boldsymbol{k_2}, \boldsymbol{k_{s3}} = \boldsymbol{S}(\varepsilon,\theta_s)\,\boldsymbol{k_3}$$

Thus the moiré wavevectors in the heterostrained WS_2/WSe_2 superlattices (Fig. S1C) become

$$\boldsymbol{K_{s1}} = \boldsymbol{k_{s1}} - \boldsymbol{k'_1}, \boldsymbol{K_{s2}} = \boldsymbol{k_{s2}} - \boldsymbol{k'_2}, \boldsymbol{K_{s3}} = \boldsymbol{k_{s3}} - \boldsymbol{k'_3}$$

We numerically fit the magnitude of $\boldsymbol{K_{s1}}$, $\boldsymbol{K_{s3}}$, and $\boldsymbol{K_{s3}}$ to find ε and θ_s. For the sample shown in Fig. 3B (also shown in Fig. S1D), the numeric fit based on the magnitude of $\boldsymbol{K_{s1}}$, $\boldsymbol{K_{s2}}$, and $\boldsymbol{K_{s3}}$ gives $\varepsilon = 0.7$ % and $\theta_s = 106.4^{\circ}$.

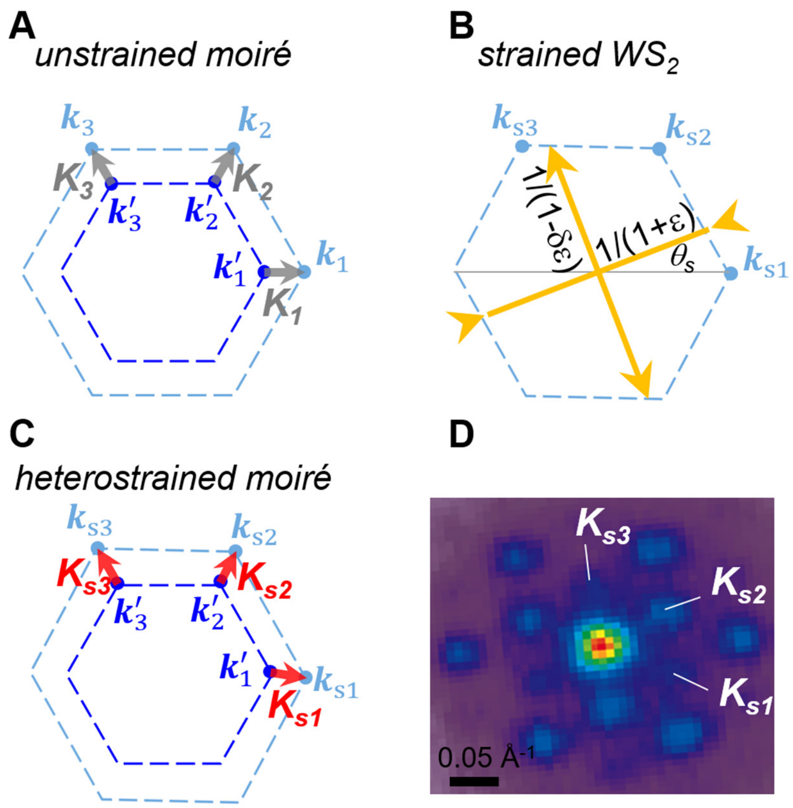

Fig. S1. Moiré wavevectors under uniaxial heterostrain. (**A**) Unstrained reciprocal wavevectors of WS_2 (light blue) and WSe_2 (blue), respectively, and the corresponding moiré wavevectors (gray). (**B**) Reciprocal wavevectors of WS_2 under a uniaxial strain of magnitude ε, along the direction θ_s with respect to x axis. (**C**) Moiré wavevectors in heterostrained WS_2/WSe_2 moiré superlattice, and the corresponding moiré wavevectors (red). (**D**) Moiré wavevectors identified in Fig. 3B for numeric fitting find ε and θ_s.

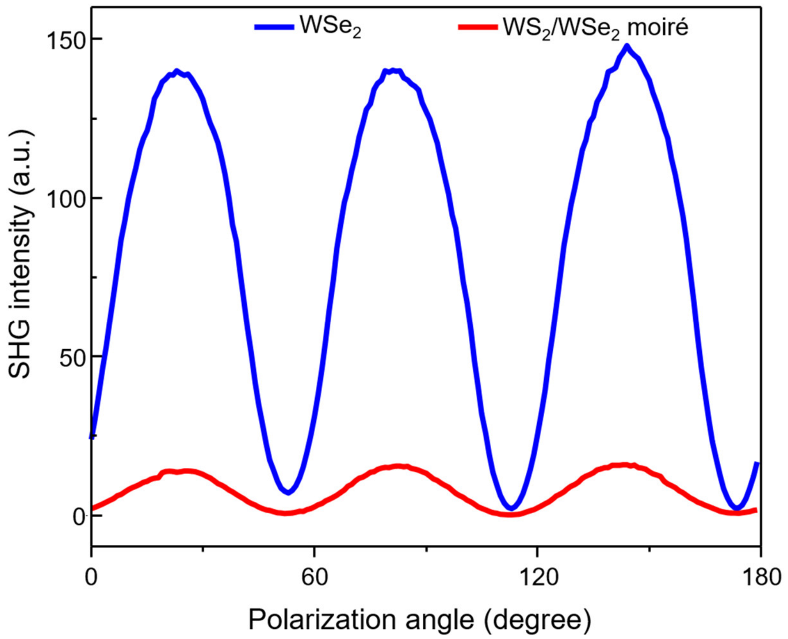

Figure S2. Polarization-resolved SHG intensity. Second-harmonic intensity as a function of the polarization angle of the excitation light taken at WSe_2 area (blue) and WS_2/WSe_2 area (red), respectively, on the sample shown in Figure 1C. The lower intensity measured from the WS_2/WSe_2 area indicates that the second-harmonic radiation from monolayer WSe_2 and WS_2 is added destructively, suggesting a 60° twist angle between WS_2 and WSe_2.
